# Vitamin A and Its Multi-Effects on Pancreas: Recent Advances and Prospects

**DOI:** 10.3389/fendo.2021.620941

**Published:** 2021-02-18

**Authors:** Yunting Zhou, Huiying Wang, Junming Zhou, Shanhu Qiu, Tingting Cai, Huiqin Li, Ziyang Shen, Yun Hu, Bo Ding, Menghui Luo, Rong Huang, Rengna Yan, Wei Xu, Cong He, Yumin Zhang, Fengfei Li, Zilin Sun, Jianhua Ma

**Affiliations:** ^1^ Department of Endocrinology, Nanjing First Hospital, Nanjing Medical University, Nanjing, China; ^2^ Department of Cadre Gastroenterology, Jinling Hospital, Medical School of Nanjing University, Nanjing, China; ^3^ Department of Endocrinology, Zhongda Hospital, Institute of Diabetes, School of Medicine, Southeast University, Nanjing, China; ^4^ Department of Endocrinology, Shenzhen People’s Hospital, The Second Clinical Medical College of Jinan University, The First Affiliated Hospital of Southern University of Science and Technology, Shenzhen, China; ^5^ Department of Endocrinology, Xuzhou Central Hospital, Xuzhou Institute of Medical Sciences, Xuzhou Clinical School of Nanjing Medical University, Xuzhou, China; ^6^ State Key Laboratory of Bioelectronics, School of Biological Science and Medical Engineering, Southeast University, Nanjing, China

**Keywords:** vitamin A, diabetes, pancreas, development, function, immune response, pancreatic stellate cells

## Abstract

Vitamin A (VA), which is stored in several forms in most tissues, is required to maintain metabolite homeostasis and other processes, including the visual cycle, energy balance, epithelial cell integrity, and infection resistance. In recent years, VA molecules, also known as retinoids, have been extensively explored and used in the treatment of skin disorders and immune-related tumors. To date, several observational and interventional studies have explored the relationship between VA status and the pathogenesis of diabetes. In particular, VA micronutrients have been shown to regulate pancreatic development, β-cell function, pancreatic innate immune responses, and pancreatic stellate cells phenotypes through multiple mechanisms. However, there are still many problems to be proven or resolved. In this review, we summarize and discuss recent and available evidence on VA biological metabolism in the pancreas. Analysis of the effects of VA on metabolism in the pancreas will contribute to our understanding of the supportive physiological roles of VA in pancreas protection.

## Introduction

The prevalence of diabetes mellitus (DM) is increasing rapidly worldwide. DM is a multifactorial disease that is typically linked to genetic information, life style and environmental stimulus (1). Nutrition metabolism, particularly most micronutrients in the organism, is also altered, either as part of the cause or effect, during the development of DM.

Vitamin A (VA), an essential nutrient that is only obtained from the diet, contributes significantly to the global health crisis affecting resource-constrained countries (2). Recent studies on the pancreas have demonstrated that VA and its receptors are directly associated with glucose metabolism (3–7). However, our understanding of the role of VA in the pathophysiological mechanisms of pre-DM and DM is still evolving. Thus, in this review, we thoroughly reviewed and summarized data regarding the influence and mechanisms of VA on endocrine function in the developing pancreas and adult pancreas.

## VA Storage in the Pancreas

VA is a term including a variety of unsaturated organic compounds, such as retinol, retinal, and retinoic acid. The predominant VA in serum is retinol, which is derived from the carotenoid, β-carotene, or from pro-VA. In addition to VA in the circulatory system, hepatic stellate cells (HSCs) account for 80% of VA storage in the body and are responsible for VA metabolic responses in target tissues (8, 9). HSCs wrap its extended tentacles around the small blood vessels formed by hepatic sinusoidal endothelial cells and exhibit a remarkable capacity for regulation of cellular contraction and blood flow (10, 11). Available evidence indicates that hepatic endothelial cells can maintain the resting state of HSCs by producing nitric oxide (12, 13). Vascular disorder caused by liver injury in which nitric oxide synthase (endothelial Nitric Oxide Synthase, eNOS) activity is weakened, can effectively promote HSC activation with concomitant disappearance of the VA-storing lipid droplets (14). Activated HSCs in turn exacerbate endothelial dysfunction, the formation of this vicious circle promotes the development of liver fibrosis (15, 16). Therefore, the interaction between hepatic endothelial cells and HSCs may affect the storage, transport, and usage of VA.

**Figure 1:**
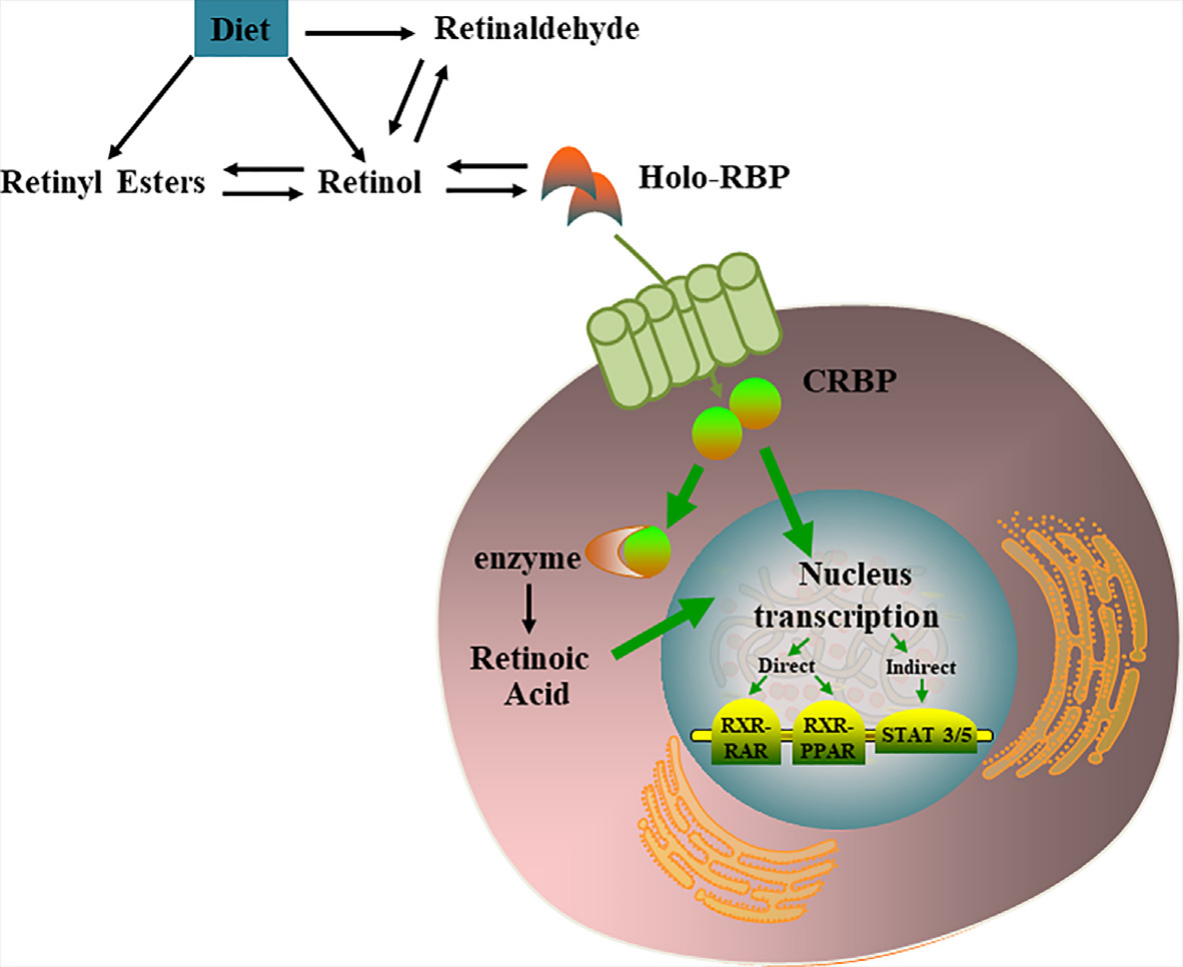
VA metabolic and signaling systems in cells. Retinol, retinal, and retinoic acid are three derivatives of VA. Each molecule has a cis and trans configuration, and the most active form is retinol. Retinol has 6 biologically active isoforms*: all-trans, 11-cis, 13-cis, 9,13-di-cis, 9-cis, and 11,13-di-cis*, with all-trans being the predominant form [21]. In cells, retinol can be converted to RA which regulates multiple nucleus transcription by activating the RXR-RAR, RXR-PPAR, STAT 3/5.

Most retinoids are stored in the liver; however, this is not the only organ involved in retinoid storage. In cells, retinol can be bound to intracellular retinol binding proteins (CRBPs), among which CRBP1 is the most abundant and widely distributed (17). Several studies have shown that specific transport proteins for retinol (RBP4) in the serum (18) and cells (19) are distributed peripherally in a circular pattern within the pancreatic islets, and their anatomical locations resemble those of α cells. The presence of these retinoid-specific transport proteins in pancreatic islets suggests that retinoids and their related proteins may be involved in the metabolism of islets, supporting the endocrine functions of islets through various mechanisms (20). VA metabolic and signaling systems in cells were shown in **Figure 1**.

Pancreatic stellate cells (PSCs) exhibit VA-specific blue fluorescence (22) and are the only cell type enriched in droplets containing retinoid in human, rat, and mouse pancreas tissues (23–25). Under physiological conditions, quiescent PSCs are abundant in droplets of retinoids composed of retinyl palmitate. However, the specific roles of retinoids in quiescent PSCs have not yet been fully established. The results from *in vitro* studies have shown that all-trans retinoic acid (AT-RA) can promote the quiescent phenotype in cultured PSCs by inhibiting the activation of α-smooth muscle actin (α-SMA) and decreasing the expression of collagen synthesis (26–28). Zhou et al. (29) found that prolonged VA deficiency (VAD) alters the phenotype of resting islet stellate cells (ISCs, the subset of PSCs) compared with that of myofibroblast-like cells with increased α-SMA expression. Moreover, reintroduction of dietary VA to VA-deficient mice restores endocrine hormone profiles and induced ISCs/PSCs to become “re-quiescent,” similar to the results observed following induction of the VA-sufficient (VAS)-controlled ISCs/PSCs phenotype. However, Trasino et al. (5) detected a decrease in the CRBP1-positive PSC population in VA-deficient mice, but did not observe increased expression of α-SMA in PSCs. There is still no thorough experimental evidence supporting for the relationship between retinoid loss and PSC activation. Nevertheless, these seemingly contradictory studies have suggested that intracellular retinoid storage in the pancreas may be a key indicator for maintaining pancreatic function by accelerating or preventing PSC activation *in vivo.*

## VA Status in Patients With Diabetes

In the context of type 1 diabetes patients (T1D), Basu et al. and Krempf et al. (30, 31) found that serum VA concentrations were significantly decreased in patients with impaired glucose tolerance (IGT) compared with those in normal individuals. In another study, serum VA levels were also decreased in young patients with T1D (32). Moreover, serum VA levels have been shown to be elevated in patients with T2D or pre-T2D, such as those with obesity and IGT (31–34). As a member of the lipocalin family of proteins, RBP4 functions together with transthyretin to transport retinol from the liver to peripheral tissues by binding to specific cell receptors (35). High serum RBP4 levels have been found to be positively associated with T2D and obesity in many human studies (36, 37). A recent meta-analysis also showed that increased RBP4 is a modest independent risk factor for women with gestational diabetes (GDM), similar to the results of case-control studies (38–41).

## The Protective Effects of VA on the Pancreas

### Effects of VA on Pancreas Development

By controlling cell specification and differentiation, VA-derived RA signals, such as the retinoid receptors, retinoic acid receptors (RAR) and retinoid X receptors (RXR), are essential for pancreatic β-cell development in the underlying endoderm (7, 42–44). RA signals imitate the directional indicator signal of the lateral mesoderm by regulating the expression of a series of growth factors and participate in the differentiation of uncommitted progenitor cells toward a pancreatic fate (45, 46). More importantly, RA can promote the formation of pancreatic duodenal homeobox-1 (Pdx1) foregut endoderm, which co-expresses pancreas transcription factor 1α (Ptf1α), a transcription factor indicative of pancreatic commitment (47). At the expense of the exocrine dorsal pancreas, Notch signaling controls early pancreatic differentiation through neurogenin 3 (Ngn3) repression, whereas RA promotes endocrine correlation with specific inhibition of Notch signaling activities (48). *In vitro*, RA also has important roles in chemical introduction protocols for induction of embryonic stem cells to differentiate into insulin-producing cells (47, 49, 50). Programming of ectodermal explants from *Xenopus* blastulae with a mixture containing RA is sufficient to drive pancreatic gene expression. The proportion of pancreatic tissue formed in such programmed explants is related to the RA concentration (51). In addition to the specific differentiation-promoting effects of RA on endocrine cells induced by stem/progenitor cell, RA can also reprogram cells to another cell type with or without reversion to pluripotent stem cells (52–54). Centroacinar cells were transdifferentiated into functional β-cells by regeneration after treatment with RA (53).

The interactions of mesenchymal-epithelial cells are necessary for proper maturation of tissues (55–57). Studies have indicated that the pancreatic mesenchyme not only influences the expansion of early pancreatic progenitors but also regulates the proliferation of terminally differentiated endocrine cells during the final phase of gestation (57). Moreover, PSCs are important mesenchymal supporting cells that can maintain the normal basement membrane to stabilize the cell cytoskeleton and structure, thereby protecting the normal function of parenchymal cells (58). Chen et al. (59) found that human fetal PSCs lost intracellular retinoid-storing lipid droplets and expressed specific activated stellate markers, α-SMA, and extracellular matrix (ECM) proteins as the cultures going on *in vitro* (e.g., collagen I, collagen IV, and fibronectin). The crosstalk between multiple integrins (β1, α3 and α5) and collagen I is essential for the cell adhesion, migration, proliferation, and growth factor production in human fetal PSCs, suggesting that human fetal PSCs may effectively regulate the ECM microenvironment required for pancreatic development. These findings initially elucidated the role of PSCs in pancreas specification induced by RA.

### Involvement of VA in Glucose Homeostasis

In a study of insulin secretion, Chertow et al. (60) found that VA-deficient puppies born from mothers with mild VAD exhibited hyperglycemia and reduced glucose-stimulated insulin secretion. Dietary VA administered in the form of RA restored euglycemia and normalized islet insulin secretion. Both dietary VAD and decreased endogenous production of RA by genetic intervention blocked RA signals in mice, leading to reductions in fasting blood glucose levels and hepatic gluconeogenesis (61, 62). Mice lacking the RA-synthesizing enzyme aldehyde dehydrogenase-1 (ALDH-1) showed lower expression levels of the key gluconeogenic enzymes, glucose-6-phosphatase and phosphoenolpyruvate carboxykinase, the latter of which is an RA-inducible target gene containing a specific RA-receptor binding site as an RA response element (61). Other findings indicated an additional mechanism through which VA affects islet function by governing islets size distribution was correlated with the α-SMA-positive ISC pool in a mouse model of dietary VAD (29).

In studies of insulin responsiveness, RBP4 has attracted much research interest in the last decade owing to its effects on insulin resistance. Basic mechanistic studies have shown that elevated serum levels of RBP4 can induce the expression of phosphoenolpyruvate carboxykinase, the key gluconeogenesis-related enzyme expressed in the liver, and further increase circulating blood glucose levels *via* increased hepatic glucose production (63, 64). Other studies focusing on target organs have shown that overexpression of adipocyte-specific RBP4 increases the levels of pro-inflammatory markers and lipases associated with lipolysis, thereby promoting insulin resistance (65). Retinol-RBP4 complex is recognized by stimulated by retinoic acid 6 (STRA6), which transports retinol from the binding protein into cells (66, 67). STRA6 can effectively weaken the insulin response because STRA6-mediated retinol transport induces receptor phosphorylation, which in turn activates the Janus kinases 2/signal transducer and activator of signal transducers and activators of transcription 3/5 (STAT3/5) activation cascade, which contributes to the expression of the STAT target gene suppressor of cytokine signaling (66, 68). Additionally, mice lacking ALDH-1 are protected from high-fat diet-induced insulin resistance, potentially because retinaldehyde can increase the expression of mitochondrial uncoupling protein 1 to drive uncoupled respiration and adaptive thermogenesis in white adipose tissue, thereby promoting the development of the brown fat phenotype, increasing energy expenditure, and suppressing body weight increases. This may also be a compensatory protection mechanism for the body. RA is the ligand of peroxisome proliferator-activated receptor δ (PPARδ) and classical RAR. RA supplementation in obese mice leads to the upregulation of PPARδ and consequent ectopic lipid deposition. Therefore, PPARδ affects lipid and glucose homeostasis, thereby enhancing the expression of insulin signaling-related genes and reducing insulin intolerance (69). Furthermore, as retinoic transcription nuclear receptors, RARβ2 agonists also dramatically reduce lipid peroxidation and oxidative stress in the pancreas of both obese and diabetic mice. This suggests that RARβ2 agonists may be useful drugs for T2D therapy and for the treatment of hepatic steatosis, which may contribute to insulin sensitivity (70).

### Effects of VA on Pancreatic Innate Immune Responses

VA and its derivatives regulate adaptive and innate immune responses through different mechanisms (71, 72). High VA levels can block the Th1 response and promote the Th2 response (73). According to studies on the effects of RA on monocytes/macrophages (74–77), RA not only suppresses the secretion of cytokines produced by Th1-type cells but also increases the secretion of cytokines produced by Th2-type cells (78, 79). Dalmas et al. (80) found that dendritic cells are endogenous RA producers in pancreatic islets. Dendritic cells in islets showed reduced ALDH activity in macrophages of interleukin (IL)-33–treated VA-deficient mice compared with mice fed a chow diet, indicating that IL-33–induced enhancement of β-cell function required VA and its conversion to RA. A similar study showed that VA exerted autoimmune protective effects in part by inhibiting CD4+CD8+ interferon (IFN)-γ-producing T cells, but had no effect on the IL-17–producing T-cell population (73, 81–83). Zunino et al. (84) demonstrated that intervention with VA dietary supplements protected against the development of T1D in mice by efficiently inhibiting the infiltration of T cells into the islets, thereby precluding the progression of insulitis and diabetes. A study by Van et al. (85) reported that ATRA-treated mice had fewer pancreatic islets and a reduced incidence of pre-insulitis, even after cell transfer with CD4+CD25+ cells, whereas mice from control group developed severe destructive insulitis. Overall, VA may have applications in the treatment of autoimmune inflammatory phenotypes to reduce the formation of autoimmune diseases, such as T1D (78, 85–88).

GDM and T2D exhibit various features associated with metabolic syndrome (89), such as obesity and low-grade inflammation (90–92). Immunologic-metabolic crosstalk also plays a role in the regulation of metabolic imbalances, which affect the immune system and obesity-associated inflammation (93). Few studies have focused on the effects of VA on the immune system in GDM and T2D. However, these data based on immunology-VA crosstalk provided us with insights into the metabolic imbalances driving GDM and T2D pathogenesis.

### Effects of VA on the PSC Phenotype

PSC activation is thought to be a key cellular event for pancreatic fibrosis in the pathological processes of serious pancreatic diseases (94). The effects of VA and its analogs on PSC activation have been reported in several studies. A treatment medium containing retinoids from activated PSCs causes phenotypic reversal to the quiescent phenotype (26–28, 95, 96). Transition of quiescent PSCs to an activated myofibroblastic phenotype is marked by profound cytoskeletal changes and elevated actomyosin contractility (97, 98). Chronopoulos et al. (27) found that ATRA impairs the capacity of PSCs to remodel the ECM to promote cancer invasion. ATRA-treated PSCs showed a marked decrease in the overall traction force generation during the early and late stages of the spreading phase and had a severely reduced ability to deform the collagen matrigel matrix, thereby confirming that ATRA treatment inhibits force generation in PSCs. Thus, ATRA treatment affected the ability of PSCs to sense extracellular mechanical cues and induces cytoskeletal changes consistent with a resting-like phenotype. Zhou et al. (29) found CRBP1 knockdown restored the polygonal appearance of quiescent ISCs, and reduced the expression of activation-related proteins, such as α-SMA and collagen synthesis, thereby producing a resting-state phenotype. Maintaining ISCs being quiescent state enhanced glucose-induced insulin release and basal insulin secretion. Thus, regulation of VA metabolism-related molecules is required to maintain a quiescent ISC population and block islet fibrosis and exocrine pancreatitis. She et al. (99) found that overexpression of sterol regulatory element-binding protein-1c in activated HSCs, which have many biological features in common with PSCs, induces a drastic reversal of the cell phenotype to quiescent HSCs. Resting HSCs contain sufficient triglycerides (100); therefore, they can be used as a source of fatty acids for esterification of retinol.

**Figure 2:**
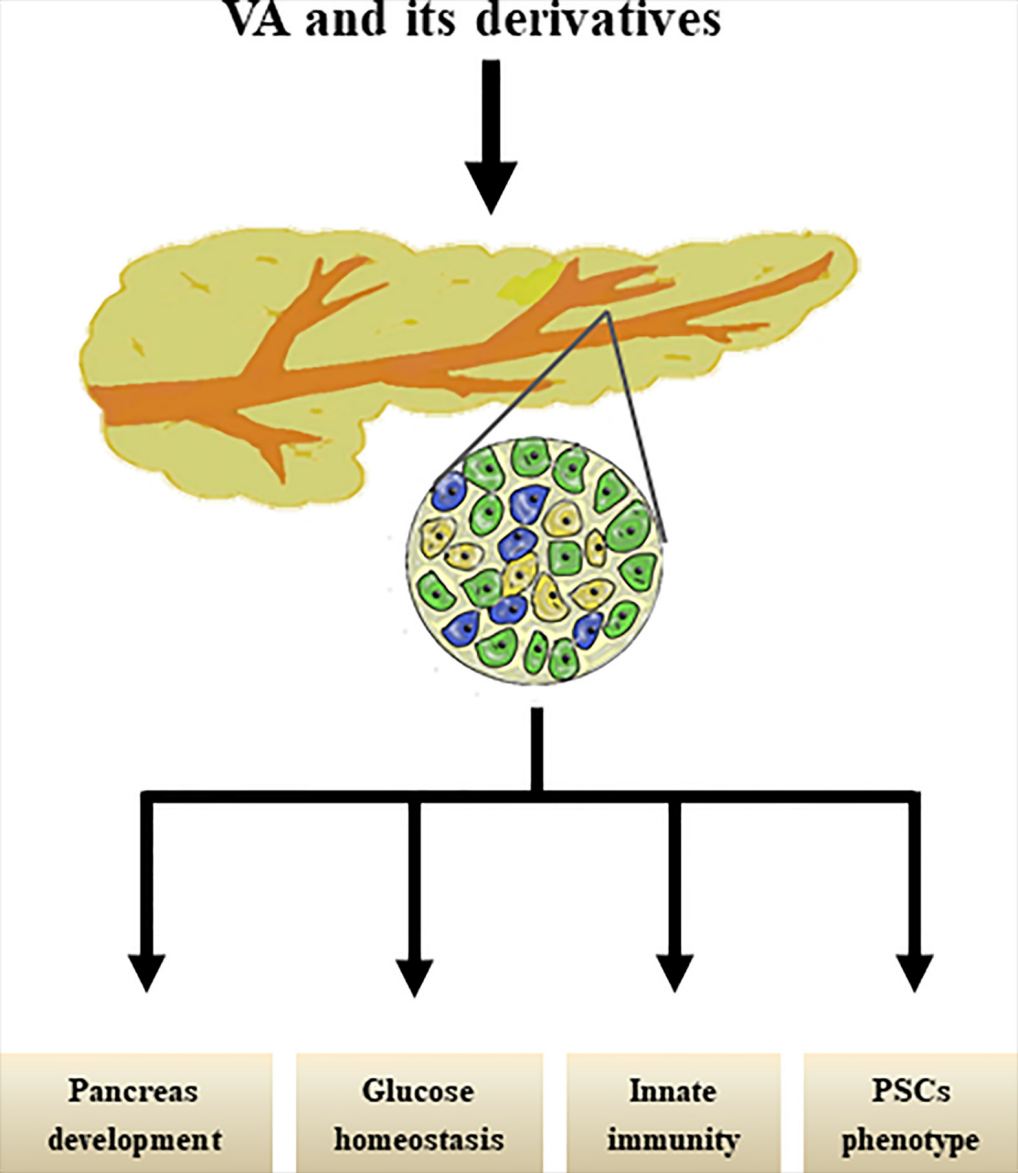
Effects of VA and its derivatives on pancreas. VA and its derivatives are reported to promote pancreas development, maintain glucose homestasis, regulate pancreatic innate immunity, and transform PSCs phenotype.

Interestingly, our group previously reviewed that PSCs share similar biological phenotypes with “universal” pancreatic stem/progenitor cells; for example, PSCs share localization, stem cell markers, signaling pathways, and multi-potential differentiation abilities with pancreatic stem/progenitor cells (101) and have therefore been proposed as a new cell type of potential adult pancreatic stem/progenitor cells. However, further studies are still needed to determine whether and how RA signals suppress the capacity of the molecule to mediate the differentiation of PSCs into pancreatic endocrine cells. The effects of VA metabolism on pancreas were shown in **Figure 2**.

## Conclusion

Based on current evidence, VA status is relevant in the pathogenesis of human DM and in the physiological processes of pancreatic development, β-cell function, pancreatic innate immune responses, and PSC phenotype. Further studies are needed to elucidate all of the physiological functions of RA, retinol, and their metabolites and to identify the mechanisms mediating the unique effects of VA on target cells and gene production.

## Data Availability Statement

All data sets generated for this study are included in the manuscript.

## Author Contributions

YtZ and HW conceived and wrote the manuscript. JZ, TC, ZyS, YH, HL, and BD collected articles. RY, RH, and ML analyzed the data. WX, CH, and FL reviewed articles. YtZ and SQ drew the figures. YmZ modified the manuscript. ZS and JM directed the manuscript. All authors contributed to the article and approved the submitted version.

## Funding

This work was supported by the National Nature Science Foundation of China (NSFC-81870563).

## Conflict of Interest

The authors declare that the research was conducted in the absence of any commercial or financial relationships that could be construed as a potential conflict of interest.
